# 阿帕替尼治疗肺原发性滑膜肉瘤术后多发转移1例

**DOI:** 10.3779/j.issn.1009-3419.2018.11.11

**Published:** 2018-11-20

**Authors:** 娣 张, 初峰 张, 其森 郭

**Affiliations:** 1 250100 济南，山东大学附属山东省肿瘤医院，山东大学 Shandong Cancer Hospital Affiliated to Shandong University, Shandong University, Jinan 250117, China; 2 250117 济南，山东大学附属山东省肿瘤医院，山东省医学科学院，山东省肿瘤防治研究院 Shandong Cancer Hospital affiliated to Shandong University, Shandong Academy of Medical Sciences, Shandong Cancer Hospital and Institute, Jinan 250117, China

**Keywords:** 甲磺酸阿帕替尼, 肺肿瘤, 原发性肺滑膜肉瘤, Apatinib, Lung neoplasms, Primary pulmonary synovial sarcoma

## Abstract

原发性肺滑膜肉瘤是一种起源于原始间叶细胞的罕见肺部恶性肿瘤，其临床特点包括生存期短、预后差，目前国内外仅有少数相关报道。近年来，靶向治疗的发展为肿瘤患者带来显著的获益。甲磺酸阿帕替尼是我国自主研发的小分子血管内皮生长因子受体-2（vascular endothelial growth factor receptor-2, VEGFR-2）抑制剂，可高度选择性地抑制VEGFR-2酪氨酸激酶活性，阻断血管内皮生长因子（vascular endothelial growth factor, VEGF）结合后的信号通路，从而强效抑制肿瘤血管的生成，目前已在多种瘤种中显示出确切的抗肿瘤效果和可接受的毒副反应。本文介绍了阿帕替尼治疗原发性肺滑膜肉瘤术后多发转移1例，以期在临床工作中为医生提供新的治疗思路。

## Abstract (en)

Primary pulmonary synovial sarcoma is a rare pulmonary malignant tumor originated from primitive mesenchymal, which has short overall survival and poor prognosis. Related case reports are lacked at home and abroad. In recent years, the development of targeted therapy has brought remarkable benefits to cancer patients. Apatinib (Hengrui Pharmaceutical Co. Ltd, Jiangsu, People's Republic of China) is a small molecule vascular endothelial growth factor receptor 2 (VEGFR-2) inhibitor, which selectively inhibits VEGFR-2 and blocks the VEGF signal pathway, then strongly inhibiting the tumor angiogenesis. Apatinib has shown favorable therapeutic effect and acceptable toxicity on various tumors. Here we report a case of primary pulmonary synovial sarcoma with postoperative multiple metastases treated with apatinib, in order to provide a new considerable treatment.

## 1 病例报告

患者男性，49岁，无吸烟史，卡氏评分为80分。2016年3月7日在查体时无意中发现右肺占位（图 1A1、图 1A2），未见明显临床症状。2016年3月9日行“右肺下叶切除+淋巴结清扫术”。术后病理示：（右肺）周围型滑膜肉瘤，肿瘤大小8 cm×7 cm×3 cm，未见神经及脉管内侵犯证据，支气管切缘未见恶性瘤细胞，肺脏破溃区残腔内查见瘤细胞，送检肺门（0/4）及气管前（0/1）、4R（0/1）、11组（0/1）、隆突下（0/1）、2区（0/3）、肺门（0/1）淋巴结未见转移性瘤细胞。免疫组化：CK（+）、EMA（+）、Vim（+）、CK19部分（+）、CD99部分（+）、Caponin部分（+）、Bcl-2弱（+）、CD34（-）、SMA（-）、S-100（-）、Desmin（-）、Ki67（+）约10%。依据美国国立综合癌症网络（National Comprehensive Cancer Network, NCCN）肿瘤学临床实践指南：软组织肉瘤（2018.v1），分期为pT3N0M0 Ⅲb期。术后未行其他治疗，定期每月复查1次。

**1:**
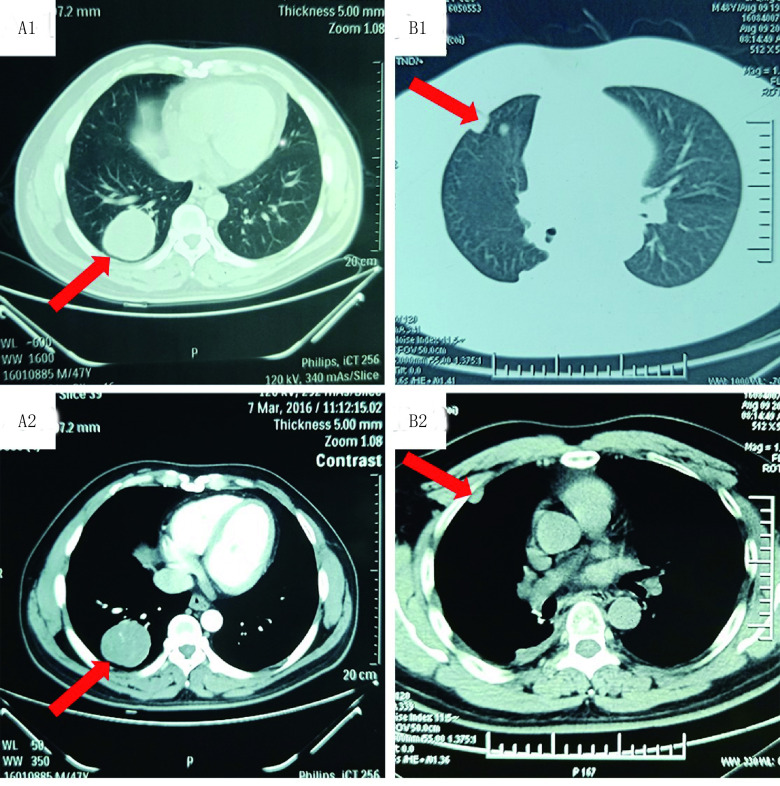
术前及术后进展的CT扫描图像：2016年3月CT扫描显示右肺占位（A1、A2）；术后定期复查，2016年8月病情进展（B1、B2）。 Chest CT scans before and after taking operation. March 2016 CT scans showed that a mass in the right lung (A1, A2); August 2016 CT images shows progression disease (B1, B2). CT: computed tomography.

2016年8月9日胸部计算机断层扫描（computed tomography, CT）示：右肺癌术后CT所见；右肺结节灶考虑转移可能性大；右侧胸腔积液、右侧胸膜肥厚（图 1B1、图 1B2）。提示肿瘤进展，修改分期为yT3N0M1 Ⅳ期。于2016年8月16日制定放疗计划，勾画右肺上叶及胸膜下高密度结节灶精准放疗，处方剂量为500 cGy/次，共11次，总剂量5, 500 cGy，期间胃肠道反应Ⅱ°，骨髓抑制0°。后予以化疗4个周期，具体方案为：异环磷酰胺2.0 g d1-3+表阿霉素120 mg d1+美司钠40 mg（异环磷酰胺后0 h、4 h、8 h），*q*21d。2个周期后CT疗效评价稳定（stable disease, SD），4个周期后复查CT提示椎旁转移，临床疗效评价为进展（progressive disease, PD），遂更换方案化疗2个周期，具体方案为：吉西他滨1.6 g d1, d8+多西他赛60 mg d1, d8，*q*21d。2017年3月7日复查胸部CT示：①右肺下叶肿瘤切除术后，右侧胸膜转移，较前略进展；②右肺上叶炎症；③右侧胸腔积液，较前变化不著；④右侧心膈角淋巴结转移，较前略增大；⑤右侧椎旁结节灶，考虑转移，较前进展（图 2A1、图 2A2、图 2A3）。

**2:**
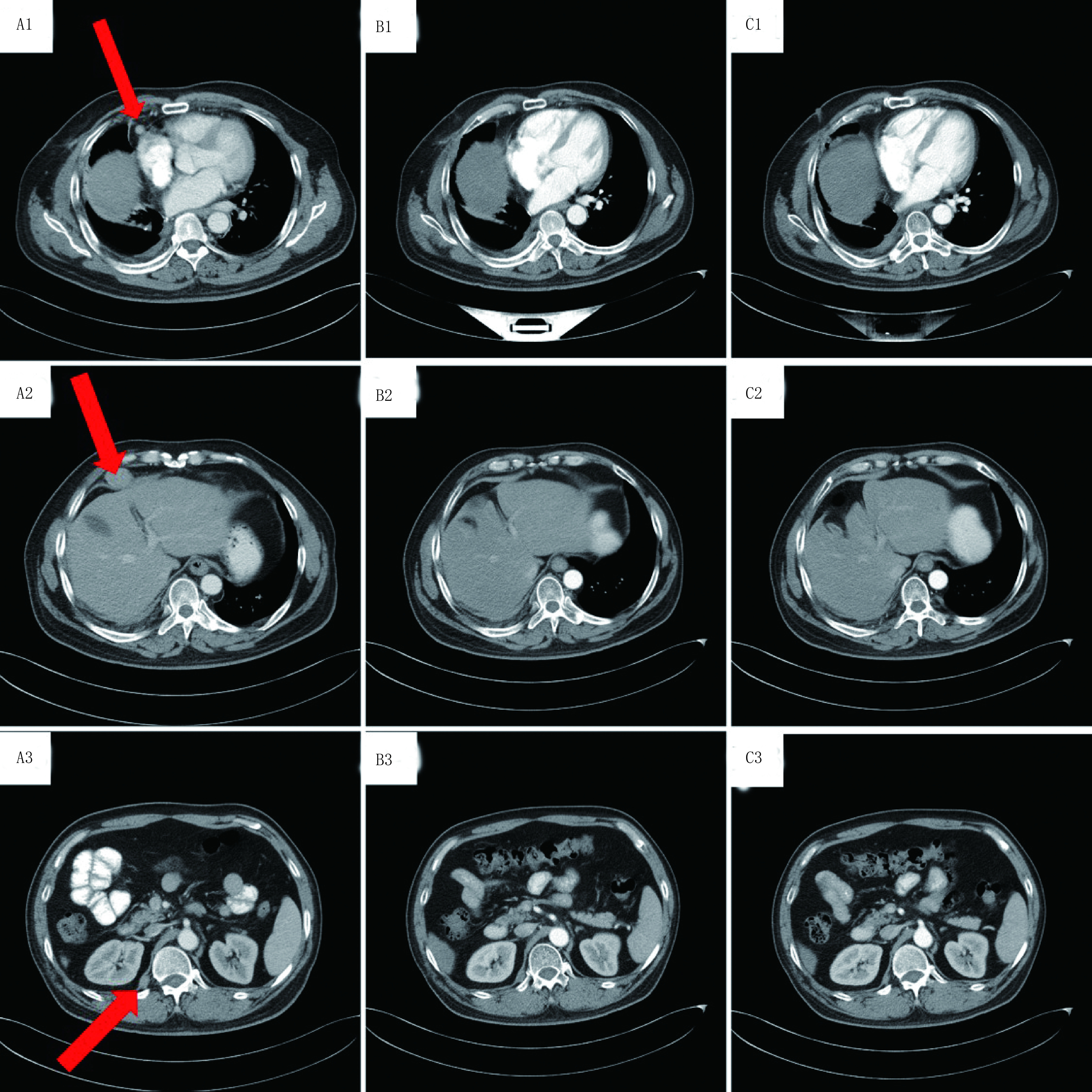
口服阿帕替尼治疗前后的CT扫描图像。阿帕替尼治疗前，2017年3月CT扫描显示右侧心膈角淋巴结转移、右侧椎旁结节灶转移（A1、A2、A3）。阿帕替尼治疗3个月后，2017年6月CT扫描显示转移灶均较前缩小（B1、B2、B3）。阿帕替尼治疗6个月后，2017年9月CT扫描显示病情稳定（C1、C2、C3）。 Chest CT scans before and after taking apatinib. March 2017 CT scans before apatinib therapy revealed metastasis of lymph node in cardiodiaphragmatic angle and right paravertebral (A1, A2, A3). After three months apatinib treatment, June 2017 CT scans showed that the metastatic nodules became smaller (B1, B2, B3). September 2017 CT images shows the condition was stable (C1, C2, C3).

患者多程治疗后病情进展，于2017年3月13日开始服用甲磺酸阿帕替尼500 mg/d，服用药物期间密切监测血压变化，并定期复查血常规、尿常规及肝肾功能，均未见明显异常。2017年6月13日CT检查：右侧心膈角淋巴结转移灶、右侧椎旁转移灶均较前好转（图 2B1、图 2B2、图 2B3）。临床疗效评价为部分缓解（partial response, PR）。2017年9月20日复查CT，临床疗效评价为稳定（图 2C1、图 2C2、图 2C3）。直至2017年12月25日复查CT病情进展（图 3）。患者行阿帕替尼靶向治疗的无进展生存期（progression-free survival, PFS）达9.4个月，耐受性较好，未出现明显不良反应。

**3:**
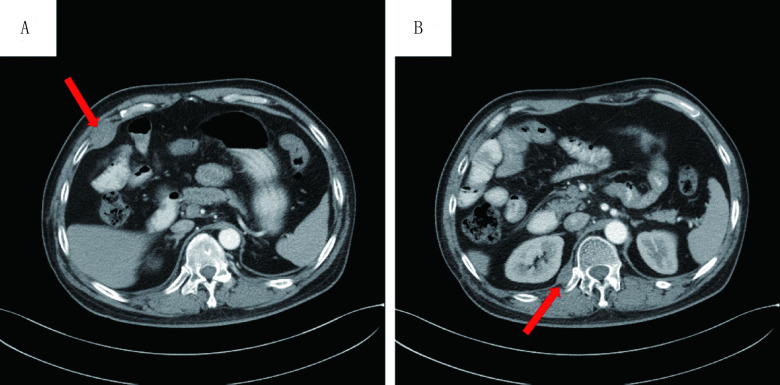
口服阿帕替尼治疗后的CT扫描图像。2017年12月CT扫描显示病情进展（A、B）。 Chest CT scans after taking apatinib. December 2017 CT scans showed that progression disease (A, B).

## 2 讨论

滑膜肉瘤（synovial sarcomas, SS）是一类源自原始间叶细胞的软组织恶性肿瘤，发病率较低，在软组织恶性肿瘤中占5%-10%。任何年龄均可发病，其中以年轻人多见^[1]^。滑膜肉瘤好发于四肢大关节附近，以下肢居多，亦可发生于无滑膜结构的部位，如头颈部、躯干、腹部、骨盆、纵隔、肺脏、心脏和肾脏等^[2]^。滑膜肉瘤在组织学上呈现出单相缩型细胞型、双相型、单相上皮型和低分化型四种类型^[3]^，其遗传学改变包括特异性t（X; 18）（p11;q11）染色体易位，并产生融合基因如*SS18*-*SSX1*、*SS18*-*SSX2*、*SS18*-*SSX4*等。

原发性肺滑膜肉瘤是一种罕见的肺部恶性肿瘤，约占肺原发性恶性肿瘤的0.5%^[4]^。原发性肺滑膜肉瘤的临床表现、影像学和实验室检查均不具有明显特异性。主要临床表现为发热、咳嗽、胸痛、咯血、呼吸困难等，易侵犯胸膜导致胸腔积液，发病早期误诊率较高^[5]^。隐匿性原发性肺滑膜肉瘤可表现为顽固性自发性气胸和大疱性病变^[6]^。部分患者也可无任何临床症状^[7]^。影像学检查提示多以周围型为主，中央型少见，影像学特点为肺内不均匀肿块，形态不规则，增强扫描强化不均匀^[3]^。其诊断有赖于结合临床、病理组织学、免疫组织化学和*SYT-SSX*融合基因检测。鉴别诊断主要包括神经内分泌肿瘤、鳞状细胞癌和各种肉瘤等^[4]^。因此，正确的诊断是行进一步治疗及改善预后的前提。

目前针对原发性肺滑膜肉瘤的治疗尚无统一方案，主要以手术治疗为主，根据临床分期选择辅助化疗和放射治疗^[5]^。放疗适宜剂量不应 < 40 Gy，放射野应包括瘤体及周围2 cm-5 cm正常组织；化疗药物临床上常用阿霉素、异环磷酰胺等^[3]^。据相关文献报道，成功的完全手术切除与存活率有一定的相关性^[8]^。经典的临床和分子预后影响因素包括晚期、肿瘤部位、肿瘤直径（> 5 cm）、年龄（> 20岁）、不完全切除、*SYT-SSX1*变异和肿瘤坏死等^[9]^。部分晚期原发性肺滑膜肉瘤患者因不能手术或已出现转移，其生存期（overall survival, OS）一般不超过1年，预后极差。本例患者无任何症状，于体检时发现，与既往文献报道相符。因原发性肺滑膜肉瘤目前没有最佳的治疗措施，对放化疗不敏感性，临床证据显示很多软组织肉瘤患者化疗无效或不能耐受化疗的毒副作用，根据病理分期、患者及家属意愿等情况综合考虑，本例患者术后未立即接受化疗，每月于当地医院复查。后因病情进展，尝试选用放化疗以延长生存期及提高预后。

恶性肿瘤的生长、侵袭和转移与肿瘤血管生成过程密切相关，抗血管生成是治疗肿瘤的重要措施之一^[10]^。甲磺酸阿帕替尼是我国自主研发的VEGFR-2抑制剂。2014年10月17日国家食品药品管理监督总局（China Food and Drug Administration, CFDA）批准甲磺酸阿帕替尼用于晚期胃癌或胃食管结合部腺癌三线及三线以上治疗。其主要不良反应为高血压，蛋白尿，血小板下降，白细胞下降，手足综合症和胆红素升高等，大部分均为轻、中度，经对症治疗后大部分可缓解^[11]^。目前已有多项研究^[12-16]^发现阿帕替尼在多种实体瘤中表现出良好的抗肿瘤能力。Li等^[16]^回顾性分析16例Ⅳ期肉瘤患者化疗失败后应用阿帕替尼治疗的疗效，结果显示客观缓解率（objective response rate, ORR）为和疾病控制率（disease control rate, DCR）分别为20.0%和80.0%，中位PFS达8.84个月，因服药所致的3级/4级不良反应主要是高血压（18.7%）、手足综合征（12.5%）和蛋白尿（6.3%）。回顾性分析64例化疗失败后的晚期骨和软组织肉瘤患者应用阿帕替尼治疗后疗效与既往研究数据接近，获得明显的疾病控制和生存获益。Zhu等^[17]^回顾性分析31例阿帕替尼单药治疗骨和软组织肉瘤的患者，研究显示ORR为33.3%，临床获益率（clinical benefit rate, CBR）高达75.0%，PFS为4.25个月，而OS为9.43个月，大多数不良反应都是1级或2级。以上研究均表明，阿帕替尼在软组织肉瘤治疗中有较高的有效性和安全性。本例患者诊断明确，原发性肺滑膜肉瘤术后多发转移治疗后进展，尝试应用阿帕替尼500 mg/d治疗，以期控制病灶进展。服药后，未观察到与阿帕替尼明确相关的不良反应，3个月后复查提示疗效PR，6个月后疗效维持SD，PFS长达9.4个月，显著延长生存。本例选择阿帕替尼作为尝试，主要归于以下几个原因：①药效学研究表明阿帕替尼可通过抑制VEGFR-2酪氨酸激酶活性，阻断VEGF与其受体结合后的信号传导，抑制肿瘤血管生成，从而治疗肿瘤^[11, 18]^。鉴于阿帕替尼对肿瘤细胞的作用机制，推测其对滑膜肉瘤疗效尚可。②对于原发性肺滑膜肉瘤的治疗尚无标准方案，目前已有较多报道证实阿帕替尼治疗晚期肉瘤的可行性，具有较好的临床应用前景。③阿帕替尼作为一种高效、低毒的抗肿瘤血管生成药物，口服给药途径方便，易为患者所接受。

目前，临床上此类原发性肺滑膜肉瘤病例报道较为少见，针对原发性肺滑膜肉瘤的治疗尚无标准方案，因此需要一种更优化的治疗选择，进一步改善患者生活质量和延长患者生存期。本例患者在治疗后疗效显著，不良反应总体可耐受并可控，提示阿帕替尼对原发性肺滑膜肉瘤具有潜在治疗价值，希望借此能为临床诊治提供一种新的治疗思路，引起医务工作者的关注。鉴于仅为个案，有待于进一步观察、探索和研究。
